# Immigration, social integration and mental health in Norway, with focus on gender differences

**DOI:** 10.1186/1745-0179-3-24

**Published:** 2007-10-30

**Authors:** Odd Steffen Dalgard, Suraj Bahadur Thapa

**Affiliations:** 1 Norwegian Institute of Public Health, Division of Mental Health, Oslo, Norway; 2 Ullevål University Hospital, Psychiatric Department, Oslo, Norway

## Abstract

**Background:**

Studies have shown that social integration may have a positive as well as a negative effect on the mental health of immigrants, depending on the social circumstances.

**Aims of the study:**

To investigate the relationship between social integration and psychological distress in immigrants in Oslo, Norway, with focus on gender differences.

**Methods:**

The study was based on data from a community survey in Oslo (N = 15899), and included 1448 immigrants from non-Western and 1059 immigrants from Western countries. Psychological distress was measured by a 10 items version of Hopkins Symptom Check List (HSCL-10), and social integration was measured by an index based on four items: Knowledge of the Norwegian language, reading Norwegian newspapers, visits by Norwegians and receiving help from Norwegians. Information on paid employment, household income, marital status, social support and conflicts in intimate relationships was also included in the study.

**Results:**

The non-western immigrants showed a higher level of psychological distress than the immigrants from western countries. In men this could be explained by the combination of less social integration, less employment, lower income, less social support and more conflicts in intimate relationships among non-western compared to western immigrants. In women the difference in level of psychological stress could not be explained by these variables, even if it was reduced. A reason for this seemed to be that social integration in non-western immigrants had a different effect on mental health in men and women. In men, social integration showed a positive effect through employment and income, as well as a positive effect in other areas. Also in non-western women social integration showed a positive effect through greater access to employment and income, but this effect was levelled out by integration causing problems in other areas.

**Conclusion:**

Unexpectedly, social integration in non-western immigrants was associated with good mental health in men, but not in women. A possible explanation for this might be that the traditional female role in these countries is more challenged by social integration into a Western country than the male role, resulting in conflicting norms, threat to the self and/or loss of identity.

## Background

It is well known that immigration may lead to poor as well as good mental health, depending on the circumstances. So called "acculturation" are among the factors which have been most widely studied in relation to immigration and mental health, but the results are rather inconclusive. By acculturation is then meant: "A 'phenomena' which results when group of individuals from different cultures come into continuous first hand contact with subsequent changes in the original culture patters of either or both groups [1]. The term has, however been used somewhat differently by different authors. Whereas Berry [2] used the term as a categorical variable, distinguishing between different modes of dealing with acculturation (assimilation, integration, separation and marginalization), other authors have used it as a continuous variable, indicating the degree of adopting the cultural values and life style of the host population [3], much in the same way as "social integration". It is in this last sense that the concept has been used in the studies referred to below.

### "Acculturation" and mental health

In some studies acculturation is associated with good mental health, in others with mental health problems. Different measures of degree of acculturation have been used in these studies, the most widely used probably being the learning of the language of the host country. Other indicators have been share of cultural values with the host population and use of host media. Several community studies in U.S. have indicated that immigrants of Hispanic ethnic origin have a lower rate of psychiatric disorder than the host population, and that the rate was increasing by increasing acculturation in terms of learning the English language [4], and by increasing length of stay in U.S[5]. The findings were somewhat surprising, given the low socio-demographic status of the immigrant groups. On this background the authors concluded that cultural factors, rather than socio-economic conditions, explained the differences in mental health. In a community sample in U.K. immigrants from India, Pakistan, Bangladesh and China had lower rates of anxiety and depression than the host population, whereas the Irish had higher rates [6]. Among the Pakistani and Bangladeshi groups who spoke fluently English, the prevalence of anxiety was doubled, whereas there was almost no difference for the Indians. Ethnic minority groups who were born in UK, or who had immigrated before the age of 11, were more likely to suffer from mental health problems, indicating that problems with acculturation develop over time, contrary to "cultural chock", which is described more like a transitional emotional reaction [7]. In a study of Greek immigrants in New York, those who were most "Americanized", in terms of sharing American values and customs, had the highest score on psychological distress [8]. In a study from a recently established neighborhood in Oslo, however, applying the same method of measuring acculturation as in the study from New York, the results were opposite [9]: The immigrants with least acculturation, in terms of sharing Norwegian customs and contact with Norwegian culture, had the highest rate of psychological distress. Likewise, in a study of Pakistani immigrants in U.K. [10], depression was associated with lack of fluency in English. Similarly, other studies have indicated that lack of proficiency in the language of the host population may lead to stress and mental health problems. In a study in Australia, for instance, poor knowledge of English, and subsequent unemployment, contributed to increased stress among immigrants [11]. In Canada Vietnamese refugees, and students from other Asian countries, had better mental health the more contacts with Canadians and Canadian culture [12]. As a conclusion then, acculturation may be associated with good as well as poor mental health, and the reasons for this are not clear.

### Recent studies of immigration and psychological distress in Oslo

In a survey in Oslo, several studies have shown increased prevalence of psychological distress among immigrants from Africa, Asia, East-Europe, whereas immigrants from western countries had about the same prevalence as the Norwegian born [13-17]. Among non-western immigrants, lack of paid employment, negative life events, lack of social support and a feeling of powerlessness were important explanatory factors for both men and women. Denial of jobs and past traumatic experiences was stronger associated with psychological distress in men, whereas older age, living without a partner and experiencing denial of housing seemed more important in women. Visits made by a Norwegian were associated with good mental health in men, but not in women [16].

### Critics of the concept of "acculturation"

Even if "acculturation" is a commonly used research variable in health studies like those referred to above, the use of the term has been heavily criticized, especially with respect to studies of US Hispanics [18]. According to this criticism, the complexity of culture, and what cultural adaptation really means, has not been taken seriously enough into consideration in these studies, and ethnic stereotyping has been prevalent. In agreement with this criticism, the term "acculturation" in the present study has been replaced by the term "social integration", even if the term has been defined in much the same way as "acculturation" in the studies referred to above. By social integration is then meant the adaptation of norms and values of the "main stream" majority culture by the minority group, especially with respect to language, daily costumes and social behavior.

### Factors influencing social integration

An important factor affecting the relationship between immigration and mental health, and between social integration and stress, seems to be the size of the immigrant community. As suggested by Murphy [19] the risk of mental health problems seems to increase by decreasing size of the immigrant group. A reasonable explanation for this is that the smaller groups, with small "ethnic density", cannot provide the same social and cultural support as the bigger groups, leaving the immigrants more vulnerable for cultural pressure from the host community. Whereas the bigger groups may establish their own ethnic subculture, and make inetgration into the host country less important, immigrants belonging to smaller groups will feel a stronger pressure towards integration.

Another factor likely to affect mental health and social integration among immigrants, is the attitudes of the host population towards the new-comers. Do they represent a pluralistic or multicultural ideology, with attendant tolerance for cultural diversity, or is there an assmilationist ideology, with pressures to conform to a single cultural standard [12]. If there is an assmilationist ideology, it is of importance how fast the new-comers are expected to assimilate, and there is some evidence that a pressure towards fast assimilation increases the risk of mental disorder, and that a pluralistic community is best for the mental health of immigrants [19].

A third factor playing an important role for social integration, is the cultural distance between the immigrants and the host population. If the cultural gap is wide, eventually with conflicting values, integration may be difficult. Especially in relation to immigration to Western countries from Asia or Africa, the conflict between individualistic and collectivist cultures is of relevance [3]. Individualistic society, then, is one where the ties between individuals are relatively loose, and everyone is expected to look after himself/herself and his/her immediate family, whereas collectivism refers to a type of society to which people from birth onwards are integrated into strong cohesive in-groups, especially family and kinship, which throughout their lifetime continues to protect them an demand unquestionable loyalty [20]. Not all individuals being alike, this means than an individualistic person from a collectivist culture may adjust easily to an individualistic society, and *vice versa*.

The possibility of gender differences in the process of social integration is barely mentioned in the literature referred to above, as if men and women of the same ethnic origin have more or less the same problems in relating to the new cultural environment. This is not necessarily so, and in a qualitative study of elderly Indian women in Canada, there are examples of cultural conflicts which seem to hit women stronger than men [21]. The traditionally strong position of the elderly women in the family, not least in the function of mother-in-law is eroded, and their inability to transmit their culture and traditions to next generation is felt as a loss and a source of sadness. Limited facility with the English language and household responsibility make it difficult for them to take part in the social life, and social isolation is likely to be a bigger problem for them than for men. Conflict with social norms, threats to the self and/or loss of identity could also be sources of distress, relatively stronger in the non-western immigrant women than men.

To which extent the contrasting associations between social integration and mental health may be explained by the factors mentioned above, is not certain. However, it seems important in future research to take them all into consideration when investigating the relationship between social integration and mental health.

### Aims of the present study

The aim of the present study is to investigate the associations between social integration and psychological distress in the same Oslo sample as referred to above. More specifically, one wish to explore to which extent differences in social integration, and other psycho-social variables, explains the differences in psychological distress between the immigrants from non-Western and Western countries, and to which extent these differences are related to length of stay in Norway.

### Hypotheses

Because of differences in cultural distance, social integration is stronger among immigrants from Western than from non-Western countries. Given the earlier findings from Oslo [9], strong integration is expected to be associated with good mental health, and social integration is an important explanatory variable between immigration and mental health.

## Methods

### Sample

The University of Oslo, National Health Screening Service of Norway (now the National Institute of Public Health), and Oslo Municipality jointly organized a general health survey known as the Oslo Health Study in 2000–2001. The study subjects were all the inhabitants of Oslo born in 1970, 1960, 1955, 1940/41 and 1924/25. The details on the methodology are described elsewhere [22]. Of the 40,888 in the sample, only 18,770 (45.9%) participated in the study. However, the weighted prevalence estimates of self-rated health and other analyzed variables differed only slightly between attendees and the target population, and the association measures were found to be less influenced by the self-selection (Søgaard et al., op cit.). In the present sample only those who had filled in information on mental health where included (N = 15899). Among those, 13392 were born in Norway, 840 in West-Europe, 225 in America, 232 in Eastern Europe, 264 in the Middle East, 218 in Africa, 453 in the Indian subcontinent and 275 in the rest of Asia.

According to cultural distance from Norway, the immigrant sample was split in two main groups: Born in West-Europe or America (north, middle and south) – "Western countries" and born in East-Europe, Asia or Africa – "non-Western countries". Even if the East-European countries are not culturally so different from Norway, they are put in the non-western group because of common differences in language, religion and political system.

### Instruments

The Oslo Health Study questionnaire was translated into the following 11 languages other than Norwegian: Albanian, Arabic, English, Farsi, Serbo-Croatian, Somali, Spanish, Tamil, Turkish, Urdu and Vietnamese. The variables used in the present study are the following:

#### Mental health

A 10-items version of Hopkins Symptom Check List [23] was used as a measure of psychological distress. Each item was rated on a scale of 1 (not at all) to 4 (extremely) during the past week. In contrast to the 25 items Hopkins Symptom Check List (HSCL-25), where symptoms can be subdivided into depression and anxiety categories [24], the HSCL-10 provides a measure of global psychological distress. The internal consistency of the scale was high in the sample (Cronbach's alpha = 0.89), and there were no difference with respect to this between the Norwegian born and the two immigrant groups. The distress score was used as a continuous variable in the analyses.

Non-responders to HSCL-10 were unevenly distributed across the migratory groups, 4, 3% of the Norwegian born, 6, 2% of the immigrants from western countries and 20, 5% of thenon-western countries being non-responders. This means that especially the last group of immigrants is substantially reduced when including HSCL-10 in the analysis.

#### Social integration

Four items were used as indicators of acculturation into the Norwegian community:

Knowledge of the Norwegian language

Reading Norwegian newspapers last year

Visit by Norwegians last year

Help/support from Norwegians last year

The first item had 5 response alternatives from "very good" to "bad", and the other items had 4 response alternatives from "daily" to "never". An index was established by summarizing the scores of each item (Cronbach's alpha: 0.57), high score meaning high acculturation.

Range 4 – 16, mean 11.42.

#### Social support

Social support was measured by one question:

How much positive interest are people showing in what you are doing

There were 5 response alternatives from "strong interest" to "no interest", and the question was intended to tap information of emotional, rather than instrumental support.

#### Recent conflicts in intimate relationships

Three items were selected from a commonly used instrument to measure negative life events during the last 6 months [25]:

You have been separated/divorced because of problems in your marriage/partnership

You have broken a longstanding relationship

You have had serious problems with close friend, relative or partner

The three dichotomous items were summarized to an index. Range 0 – 3, mean 0.22.

#### Socio-demographic variables

Information on family income was gathered from register data.

Information about employment was based on the question:

Are you in paid employment?

The response alternatives were dichotomised into to two groups: "In full-time or part-time paid employment", "not in paid employment".

Marital status was dichotomized in married/cohabitant and the rest.

#### Length of stay in Norway

The immigrant sample was split in three groups according to length of stay in Norway:

1–9 years, 10–19 years, 20 years or more.

### Statistical analyses

The associations between psychological distress (HSCL-10) and place of birth, length of stay in Norway, social integration and social network were estimated by General Linear Model analyses, presenting marginal means and 95% confidence interval. The same method was used to estimate the associations between place of birth, social integration and network variables. Multiple linear regression analyses were used to estimate the associations between psychological distress (HSCL-10) and place of birth, when adjusting for various predictors, presenting standardized Beta coefficients and level of significance. Data were analyzed by Statistical Package of Social Sciences (SPSS), version 12.

## Results

### Place of birth and psychological distress

The associations between place of birth and psychological distress are shown in table 1. The Norwegian born had the lowest rate of psychological distress. Immigrants from non-Western countries had a significantly higher rate than the Norwegian born and the Western immigrants. The last group had a slightly increased rate of psychological distress compared to the Norwegian born (statistically significant only in men). When splitting the sample in 8 regions according to birth, the immigrants from Middle-East had the highest prevalence of psychological distress (1.86), whereas the immigrants from East-Europe, Africa, the Indian Subcontinent and the rest of Asia were on about the same level (1.50–1.55). For the western immigrants the rates were respectively 1.33 and 1.37 (all scores adjusted for age and gender).

**Table 1:** Psychological distress (HSCL-10) by gender and place of birth. Means, adjusted for age

Place of birth	Men N=	HSCL-10		Women N=	HSCL-10		Total
					
		Mean	95%CI		Mean	95%CI	
Norway	6036	1.24	1.23–1.25	7356	1.37	1.36–1.38	13992
Western countries	420	1.31	1.26–1.35	639	1.40	1.34–1.42	1059
Non-western countries	720	1.53	1.47–1.58	728	1.59	1.56–1.68	1448

### Place of birth, length of stay in Norway and psychological distress

The rates of psychological distress according to length of stay in Norway are shown in table 2. For all immigrant groups, except for non-western men, the level of psychological distress seemed to increase by increasing length of stay in Norway. In non-western men, however, the trend was opposite. The differences when splitting by length of stay were not statistically significant, but there was a marginally significant interaction between gender and length of stay among non-western immigrants (p = 0.05) with respect to psychological distress.

**Table 2:** Psychological distress (HSCL-10) by gender, place of birth and length and stay in Norway. Means, adjusted for age

	Place of birth	Length of stay in Norway	HSCL-10	95% CI
Men	Western countries	1–9 years	1.20	1.04–1.37
		10–19 years	1.22	1.07–1.38
		20 years or more	1.25	1.14–1.36
	Non-Western countries	1–9 years	1.69	1.50–1.88
		10–19 years	1.52	1.36–1.68
		20 years ore more	1.49	1.35–1.63
Women	Western countries	1–9 years	1.36	1.21–1.51
		10–19 years	1.39	1.24–1.53
		20 years ore more	1.43	1.33–1.53
	Non-Western countries	1–9 years	1.56	1.38–1.75
		10–19 years	1.59	1.41–1.77
		20 years ore more	1.67	1.47–1.87

### Place of birth and socio-demographic variables

The two immigrant groups differed significantly with respect to socio-economic status, like household income and paid employment. Whereas 34% of the non-western immigrants belonged to the lowest income group, the comparable figure for western immigrants was 11%. Among the non-western immigrants 34% were without paid employment compared to 15% among the immigrants from western countries. With respect to marital status, 72% of the non-western immigrants were married/cohabitant, compared to 49% of the western.

### Place of birth, social integration and social network characteristics

As shown in table 3, all four indicators of social integration were least favourable for the non-western immigrants, and the social integration index showed that the non-western immigrants were significantly poorer integrated than the western immigrants. Also with respect to social support and conflicts in intimate relationships, the scores were less favourable for non-western than for western immigrants. For social support the scores were respectively 3.87 and 3.62 for men (p = 0.001), and 3.95 and 3.77 for women (p = 0.005). For conflicts in intimate relationships the scores were respectively 0.23 and 0.28 for men, and 0.20 and 0.37 for women. Only for women the difference was significant (p < 0.001).

**Table 3:** Indicators of social integration by place of birth and gender. Means, adjusted for age

Indicators of social integration	Place of birth	Men	Women	Total
Knowledge of Norwegian language	Western countries	3.14 ***	3.39 ***	3.30 ***
	Non-western countries	2.74	2.68	2.74
Reading Norwegian newspapers	Western countries	3.62 ***	3.59 ***	3.62 ***
	Non-western countries	3.29	2.93	3.13
Visit by Norwegians	Western countries	3.02 ***	3.10 ***	3.07 ***
	Non-western countries	2.22	2.35	2.28
Help/support from Norwegians	Western countries	2.59 ***	2.73 ***	2.67 ***
	Non-western countries	1.80	1.93	1.86
Social integration index	Western countries	12.51***	13.22 ***	12.92 ***
	Non-western countries	10.33	10.78	10.56

P < 0.05 *

P < 0.01 **

P < 0.001 ***

### Social integration and psychological distress

The relationship between social integration and psychological distress in the various groups is shown in table 4. Social integration was then split in three groups of about equal size.

**Table 4:** Psychological distress (HSCL-10) by gender, place of birth and social integration. Means, adjusted for age

	Place of birth	Social integration	Means	95% CI
Men	Western countries	High	1.29	1.19–1.40
		Medium	1.12	1.00–1.25
		Low	1.38	1.15–1.62
	Non-western countries	High	1.32	1.13–1.51
		Medium	1.46	1.32–1.60
		Low	1.68	1.53–1.83
Women	Western countries	High	1.34	1.24–1.44
		Medium	1.40	1.28–1.52
		Low	1.46	1.18–1.73
	Non-western countries	High	1.67	1.46–1.88
		Medium	1.65	1.45–1.85
		Low	1.58	1.41–1.76

For all groups, with the exception of women from non-Western countries, those with lowest social integration had the highest level of stress (significant only for non-western men). In non-western women, however, the trend was opposite, with the highest level of psychological distress among those with highest integration. This gender difference among the non-western immigrants was the same for each of the four items in the index of integration (data not shown).

### Do differences in psychosocial and socio-demographic variables explain the differences in psychological distress between the two immigrant groups?

To investigate this, multiple linear regression analyses have been carried out, with psychological distress as dependent variable, and place of birth as independent variable, with adjustment for various combinations of predictors (table 5). The associations between the predictors and psychological distress are given by standardized *beta coefficients*. For place of birth, positive coefficient means that that the risk of psychological distress is higher in the non-western than the western group. In model 1, with adjustment only for age, the results were similar in both genders: the level of psychological distress was significantly higher in non-western than western immigrants. In the further analyses, however, the associations were different in men and women. In men, the *beta *for place of birth was reduced when adjusting for social integration (model 2), whereas this was not the case in women. When adjusting for household income, employment and marital status (model 3), the *betas *for place of birth were reduced in both genders. When adjusting for social integration, in addition to the work-related variables and marital status (model 4), the effect was opposite in men and women. In men, this adjustment reduced the *beta *for place of birth further, whereas the opposite was true in women. In the last model, with adjustment also for social support and conflicts in intimate relationships (model 5), the *beta *for place of birth was reduced in both genders, in men to a level below statistical significance. In women, however, the *beta *was still significant, indicating that the non-western women had a higher level of psychological distress than the western women, even when adjusting for all these variables.

**Table 5:** Multiple regression analysis. Dependent variable: HSCL-10. Independent variables: Various predictors

Gender	Predictors	Standardized beta coefficients				
		
		Model 1	Model 2	Model 3	Model 4	Model 5
Men	Place of birth	0.17 ***	0.09	0.08 *	0.05	0.02
	R Square	0.03	0.05	0.13	0.13	0.17
Women	Place of birth	0.18 ***	0.19 ***	0.11**	0.16 ***	0.12 *
	R Square	0.03	0.04	0.09	0.10	0.20

Model 1: Adjusted for age

Model 2: Adjusted for social integration

Model 3: Adjusted for household income, unemployment and marital status

Model 4: Adjusted for social integration, household income, unemployment and marital status

Model 5: Adjusted for social integration, household income, unemployment, marital status, social support and conflicts in intimate relationships

To investigate further the gender differences in the non-western immigrants, multiple regression analyses were done for this group separately, with adjustment for different combinations of predictors (table 6). The patterns were clearly different in men and women:

**Table 6:** Immigrants from non-Western countries. Multiple linear regression analyses. Dependent variable: Psychological distress (HSCL-10). Independent variables: Various predictors, adjusted for age

Gender	Predictors	Standardized B coefficients			
		
		Model 1	Model 2	Model 3	Model 4
Men	Social integration	-0.19 ***		-0.10	-0.08
	H.h. income		-0.15 **	-0.12	-0.06
	Unemployment		0.27 ***	0.27 ***	0.22 ***
	Marital status		-0.03	-0.05	-0.02
	Social support				-0.25***
	Confl.intim.rel				0.15 *
	
	R Square	0.04	0.13	0.13	0.20

Women	Social integration	0.03		0.09	0.09
	H.h. income		-0.10	-0.16 *	-0.11
	Unemployment		0.17 **	0.15 *	0.15 *
	Marital status		-0.18 **	-0.15 *	-0.04
	Social support				-0.18 **
	Confl.intim.rel				0.25 ***
	
	R Square	0.02	0.10	0.11	0.17

### Men

When adjusting for age (model 1), social integration was strongly associated with level of psychological distress. Also household income and unemployment were associated with the level of distress, income reducing and unemployment increasing the level (model 2). Marital status was not associated with distress. When adjusting for all four variables (model 3), the *betas *for social integration and household income were reduced below the level of significance, whereas the *beta *for employment stayed the same. In the full model (model 4), employment, as well as social support and conflicts in intimate relationships were significantly associated with the level of psychological distress, whereas the *betas *for all the other variables were slightly reduced from model 3.

### Women

Differently for men, and in accordance with table 4, there was no association between social integration and level of psychological distress (model 1). Also differently from men, the *beta *for household income was not significant, whereas the *betas *for unemployment and marital status were significant (model 2). When adjusting for all four variables (model 3), the *beta *for social integration increased, opposite the trend in men. The *beta *for unemployment also increased, whereas the *betas *for the other variables showed little change. In the full model (model 4), parallel to the findings in men, only the *betas *for unemployment, social support and conflicts in intimate relationships were significant.

## Discussion

The findings of the study were partly in agreement, partly in disagreement with the hypotheses of the study. As expected, immigrants from countries culturally distant from Norway (non-Western countries) were less integrated than those from countries culturally more close to Norway (Western countries). In men from non-Western countries, the level of psychological distress was highest among those who had arrived to Norway most recently, whereas the trend was opposite for the other groups. As expected, there was a negative association between social integration and psychological distress in men from non-Western countries, but, unexpectedly, this was not the case in women. Consequently, when integration was adjusted for in the analyses, the association between non-Western countries and psychological distress was reduced in men, but not in women.

The finding that the effect of social integration was reduced in men when adjusting for paid work and household income, whereas the effects of the work-related variables showed little change when adjusting for integration, may indicate that the effect of integration was partly mediated by the work-related variables: The positive effect of social integration on mental health was to a considerable extent because of social integration increasing the probability of being in paid employment, which by itself was positive for health. The findings in women were more complex. The fact that social integration in women seemed to increase the level of psychological distress, when adjusted for work-related variables, but showed little association with distress without this adjustment, indicates that a negative effect of integration in other areas was levelled out by a positive effect of being in paid work, which also in women was positively associated with integration.

There may be several reasons for the different effects of social integration in men and women from non-Western countries. One possibility is that women, with their central role in the family, to a greater extent than men are challenged by cultural values different from their own. It is not unlikely that the conflicts between the collectivistic values in most of the non-Western countries and the individualistic values in the Western countries [3] are more strongly felt by immigrant women, and stronger the more contact with the Norwegian community. It is in accordance with this that psychological distress in the present study seems to increase by length of stay in Norway for women, but not for men. Another explanation may be that social integration in women in some instances is met by negative sanctions from men of their own ethnic group. It is well known, that for instance muslim women are not always stimulated by their families to adopt the Norwegian language and Norwegian costumes, rather to the contrary, and are expected to stay apart from the Norwegian community. All together, the attempt of these women to integrate in the Norwegian community may easily lead to conflict with social norms, threats to the self and/or loss of identity, which could be a burden on mental health.

The finding that social support and conflicts in intimate relationships were strongly associated with psychological distress, and contributed to the difference in mental health between non-western and western immigrants, were in accordance with prior findings from the same sample [13], and confirms that lack of social support and interpersonal conflicts increase the risk of mental disorder, and that migration, especially between markedly different cultures, may be interruptive to social ties.

Why do the immigrants from culturally distant countries in Oslo have an increased prevalence of psychological distress, when this is opposite of the findings from some other countries? [4-6] One possible explanation is that the immigrant groups in Oslo are relatively small, even if the total amount of immigrants is quite big (ca. 20%), and not able to provide the immigrants with a strong supportive subculture which could have a protective effect on their mental health [13]. The relatively weak social support, and the conflicts in intimate relationships, supports this.

Another possible explanation is that Norway is an assimilationistic, rather than a pluralistic country [12]. This may be because of cultural traditions in Norway, which up to recently used to be a culturally homogenous country, but also because of the rather small size of the immigrant groups, and their up till now small cultural impact on the Norwegian community. In Norway there is a strong pressure on immigrants to adopt Norwegian language and Norwegian customs, in spite of the public policy being "integration" rather than "assimilation". Even if the immigrants may keep important elements of their own culture if they are not in open conflict with the Norwegian culture, they are expected to adjust rather quickly to the Norwegian society. Over time this may have a positive effect on the mental health, but in the short run it is likely to create stress and mental health problems among those who are least able to acculturate.

A last possible explanation of the relatively high rate of psychological distress among the non-western immigrants in Oslo, could be that these immigrants simply come from countries with high rates of psychological distress. The opposite might be true for other immigrant groups, like the Hispanics in US. To investigate this further, a study of the mental health of people in Pakistan, which is one of the main providers of non-western immigrants to Norway, is carried out with the same instruments as used in the Oslo study.

The relationship between social integration, paid employment and mental health, as shown by the men in the present study, may also throw light on the different associations between integration and mental health in different countries, as referred to in the introduction. It is for instance not unlikely that the big groups of Mexican immigrants in California [5] and of Greeks in New York [8] are able to get paid work, without speaking English. Their cultural identity is well preserved within their immigrant community, and integration into the American community may then represent more of a burden than a way to a better life, at least in the short run.

From a methodological point of view, it is positive that the study was based on a fairly big sample, with information on a number of variables relevant for the immigrant situation. On the other side, the low response rate may question the respresentativeness of the sample. Even if analyses indicated that the representativeness of the total sample was good [22], it is more problematic with the immigrants, especially those from non-western countries, since their response rate to the questions on psychological distress was lower that in the total sample. Even if this makes it difficult to draw conclusions about the prevalence of psychological distress among the immigrants, this should not to the same extent affect the internal comparisons within the immigrant population, which is the aim of the study. Another weakness of the study is the cross-sectional design, which makes it difficult to draw conclusions of causality. This means that some of the conclusions are rather hypothetic, and should be tested in longitudinal studies.

## Conclusion

In men from non-Western countries social integration was positively associated with mental health, whereas this was not the case for women. This may be because of the traditional social roles of women from non-Western countries are more challenged by integration into the western culture than is the case for men from these countries. This difference should be kept in mind when dealing with integration policy towards this group of immigrants.

## Competing interests

The author(s) declare that they have no competing interests.

## Authors' contributions

OSD has designed the study, analysed the data, drafted the manuscript and approved the final version to be published. SBT has contributed to the design of the study, the analyses of data, the drafting of the manuscript and have approved the final version to be published.
